# Bioluminescent imaging in induced mouse models of endometriosis reveals differences in four model variations

**DOI:** 10.1242/dmm.049070

**Published:** 2021-08-31

**Authors:** Ashley Dorning, Priya Dhami, Kavita Panir, Chloe Hogg, Emma Park, Gregory D. Ferguson, Diane Hargrove, James Karras, Andrew W. Horne, Erin Greaves

**Affiliations:** 1 Medical Research Council Centre for Reproductive Health, Queen's Medical Research Institute, The University of Edinburgh, Edinburgh EH16 4TJ, UK; 2 Centre for Early Life, Warwick Medical School, University of Warwick, Coventry CV4 7AL, UK; 3 Ferring Research Institute, 4245 Sorrento Valley Blvd, San Diego, CA 92121, USA

**Keywords:** Endometriosis, GnRH antagonist, Lesion, Pain

## Abstract

Our understanding of the aetiology and pathophysiology of endometriosis remains limited. Disease modelling in the field is problematic as many versions of induced mouse models of endometriosis exist. We integrated bioluminescent imaging of ‘lesions’ generated using luciferase-expressing donor mice. We compared longitudinal bioluminescence and histology of lesions, sensory behaviour of mice with induced endometriosis and the impact of the gonadotropin-releasing hormone antagonist Cetrorelix on lesion regression and sensory behaviour. Four models of endometriosis were tested. We found that the nature of the donor uterine material was a key determinant of how chronic the lesions were, as well as their cellular composition. The severity of pain-like behaviour also varied across models. Although Cetrorelix significantly reduced lesion bioluminescence in all models, it had varying impacts on pain-like behaviour. Collectively, our results demonstrate key differences in the progression of the ‘disease’ across different mouse models of endometriosis. We propose that validation and testing in multiple models, each of which may be representative of the different subtypes/heterogeneity observed in women, should become a standard approach to discovery science in the field of endometriosis.

## Abstract (precis)

**Summary:** Different versions of syngeneic mouse models of induced endometriosis exhibit disparities in chronicity and cellular composition of lesions, as well as endometriosis-associated hyperalgesia.

## INTRODUCTION

Endometriosis is an enigmatic incurable condition impacting 190 million women worldwide during their reproductive years. It is associated with debilitating pelvic pain, dysmenorrhea, dyspareunia and infertility (Zondervan et al., 2020). Despite its high prevalence and devasting impact on health-related quality of life, treatment options for endometriosis remain limited, and the current main therapies for treating symptoms are surgical excision of lesions or suppression of ovarian hormones (Giudice and Kao, 2004). Recurrence is a key problem following surgical excision and both options have unwanted side effects. There is an evident unmet clinical need for new medical therapies for the treatment of endometriosis.

Endometriosis is defined by the presence of endometrial-like tissue explants, usually within the pelvic cavity, known as ‘lesions’. Endometriotic lesions contain endometrial-like stromal cells with or without epithelial glands, can be highly infiltrated by immune cells and are vascularized and innervated (Greaves et al., 2017a). Lesions can also contain a significant fibrotic component (Vigano et al., 2018). Clinically, the histological appearance of endometriotic lesions is significantly heterogeneous, which can add complexity to diagnosis (Clement, 2007). Careful reappraisals of the pathology of endometriosis highlight the variable nature of their components and suggest that typical endometriosis should contain endometroid epithelium, or stroma, or fibrosis or hemosiderin-laden macrophages. These often co-occur but not in all instances (Clement, 2007; Vigano et al., 2018). One of the most widely accepted theories for the development of endometriosis is dissemination of endometrial fragments resulting from retrograde menstruation (Sampson, 1927). However, it remains unknown why endometriosis occurs in only a minority of women when ∼90% of women experience retrograde menstruation. Heterogeneity in disease severity and lesion subtypes (superficial peritoneal, ovarian endometrioma and deep infiltrating) suggest that endometriosis could have multiple origins, and other theories are increasingly being discussed (Sourial et al., 2014).

Endometriosis only develops spontaneously in humans and some primates (MacKenzie and Casey, 1975); however, mice are most frequently used as a model for discovery science and testing potential therapeutics because of their economic viability and ease of manipulation. Many variations of mouse models of induced endometriosis are prevalent in the research community, and the only unifying characteristic of these models is the establishment of ectopic endometrial tissue. Mouse models of induced endometriosis can be classified as autologous (autotransplantation), heterologous (human endometrium xenografted into immunodeficient mice) or syngeneic (donor-recipient of same strain), as recently reviewed (Simitsidellis et al., 2018; Greaves et al., 2017a, 2020). The most commonly used mouse model is the syngeneic model as this allows the use of transgenics as donor or recipients, facilitating investigation of donor/recipient-derived cells and genes in the pathogenesis of endometriosis. The majority of syngeneic models are based on the transplantation of uterine material to ectopic locations within the pelvic cavity; however, the nature of the donor uterine material and method and site of transplantation, as well as the hormonal status of recipient mice, differs between research groups. Advantages and disadvantages of each variant have been summarized previously (Greaves et al., 2017a) but this extreme heterogeneity and lack of standardization among endometriosis models likely contributes to diminished reproducibility and comparability across studies. Importantly, translation of preclinical endometriosis studies to clinical trials has been overwhelmingly unsuccessful, and there is a distinct lack of new drugs in the pipeline (Guo, 2014; Guo and Groothuis, 2018). This very clearly highlights that we still have an insufficient understanding of endometriosis aetiology and whether preclinical mouse models effectively recapitulate pathology and symptomology in women.

The impact of interventions on endometriosis lesion size and number is improved by non-invasive imaging of lesions. This facilitates the collection of data before, during and after drug treatment during longitudinal studies, improves experimental design and reduces the number of mice required. Some groups have used fluorescent or bioluminescent imaging to aid the identification and quantification of lesion number and size (Fortin et al., 2003; Wang et al., 2014). With fluorescent models, subcutaneous placement offers preferential signal detection – only a weak signal was detected from endometrium grafted intraperitoneally (Martinez et al., 2019a). Another syngeneic model, in which donor uterine fragments from mice that ubiquitously express luciferase (UbC-Luc) were sutured to the peritoneal wall of wild-type mice, allowed robust imaging of endometriotic lesions (Becker et al., 2006). The group demonstrated that bioluminescent signal correlated well with lesion size (Becker et al., 2006).

We previously developed a mouse model of induced endometriosis that attempts to recapitulate the process of retrograde menstruation; endometrial breakdown is induced in a ‘menses’-like event (Cousins et al., 2014) in a donor mouse and the resulting ‘menstrual’ tissue is injected into the peritoneal cavity of ovariectomized recipient mice supplemented with oestradiol. Lesions form that phenocopy features of lesions recovered from women (Greaves et al., 2014). Mice with induced endometriosis also exhibited alterations in sensory behaviour and associated molecular adaptations in the nervous system (Greaves et al., 2017b). It has since been demonstrated that endometriosis lesions can be established using a minimally invasive model, in which endometrium collected from naïve mice is injected into the peritoneal cavity of intact recipients (Dodds et al., 2017). Dodds et al. (2017) compared the impact of oestrus stage on lesion development and investigated differences in the appearance and architecture of lesions across different strains of mice. However, there has been no direct comparison drawn between endometriosis mouse models. It remains unknown exactly how different models compare in terms of efficacy of endometrial tissue attachment and lesion longevity, cellular composition of lesions, development of endometriosis-associated hyperalgesia and most importantly, response to drug treatment. The minimally invasive model also negates the requirement for surgical shams and has been proposed as a preferable model for the study of endometriosis-associated pain. Although no behavioural studies have been published using this model as yet, subtle changes in spinal glia were observed (Dodds et al., 2019) that would likely be precluded in surgical models. Another similar non-surgical model used full thickness uterine tissue and the mice exhibited abdominal mechanical allodynia and spontaneous abdominal pain, as well as changes in thermal selection behaviour (Fattori et al., 2020).

In the current study, our aim was to integrate non-invasive bioluminescent imaging into our mouse model of induced endometriosis and to investigate how four different versions of endometriosis mouse models compare to one another. We compared lesion histology and bioluminescent signal, and the development of mechanical hyperalgesia in each model, as well as response to the gonadotropin-releasing hormone (GnRH) antagonist Cetrorelix.

## RESULTS

### Integration of non-invasive bioluminescent monitoring of lesions in the ‘menses’ model of endometriosis

**Fig. 1.**
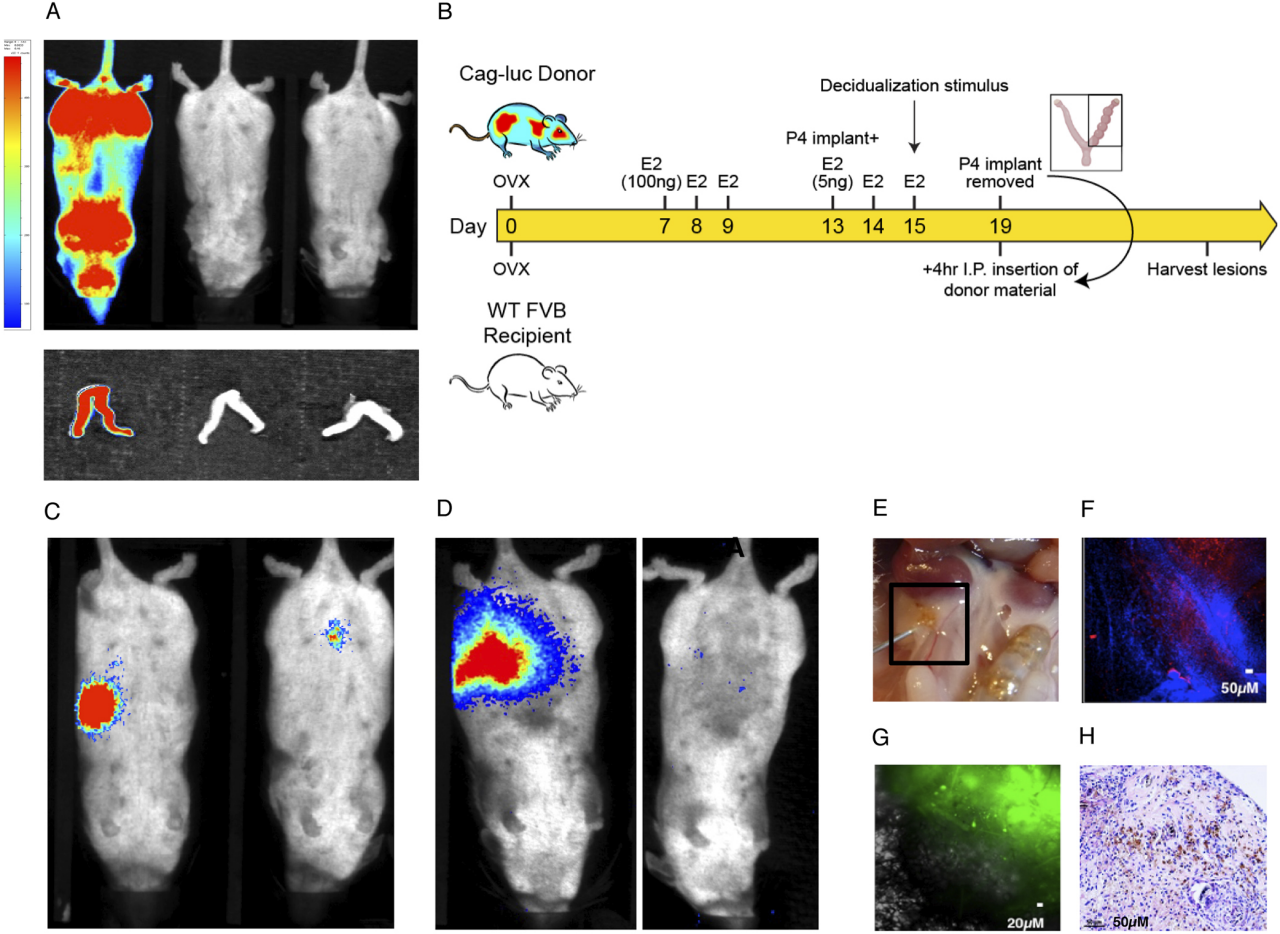
**Establishment of non-invasive bioluminescent imaging in the ‘menses’ model of induced endometriosis.** (A) Left to right: Cag-luc-eGFP mouse injected s.c. (1.5 mg in 100 µl volume) with luciferin; Cag-luc-eGFP mouse with no injection; and wild-type FVB/N mouse injected s.c. with luciferin. Whole-body imaging (top panels) and dissected uteri from corresponding mice (bottom panels). (B) Schematic representation of the DO model of endometriosis. ‘Menses’ endometrium from Cag-luc-eGFP mice was injected i.p. into ovariectomized (ovx; with add-back oestradiol) wild-type FVB/N recipient mice. Whole-body bioluminescent imaging was performed 21 days post tissue injection. (C) Whole-body images showing that bioluminescent focal lesions were localized to the abdominal region of the mice. (D) Whole-body images demonstrating the difference between i.p. (left panel) and s.c. (right panel) administration of luciferin at day 7 post tissue inoculation (in the same mouse), indicating that s.c. administration can differentiate between attached explants and unattached floating endometrial tissue. (E) At dissection, lesions were observed attached to the abdominal wall, fat and organs. (F) Paraformaldehyde (4%)-fixed lesion tissue was stained for luciferase (red), with nuclei stained with DAPI (blue). (G) Endogenous GFP (green) expression by lesions was visualized by live fluorescence microscopy prior to fixation. Surrounding GFP^−^ tissue can be observed (phase white). (H) Representative H&E image of a lesion with stromal cells with or without glandular epithelia and the presence of hemosiderin. Bar on left hand side of A (also applies to C,D) is lookup table (LUT) representing photon counts. Set to 0.0653 (minimum) and 0.46 (maximum) ×10^-3^ counts.

Luminescence is ubiquitous in homozygous CAG-luc-eGFP mice following subcutaneous (s.c.) administration of 1.5 mg luciferin. Images were acquired using a Biospace PhotonIMAGER (Fig. 1A, top panel). Bioluminescence was also confirmed in dissected uteri (Fig. 1A, bottom panel). To allow non-invasive bioluminescent detection of endometriosis ‘lesions’ in our established model, endometrial tissue from donor CAG-luc-eGFP mice manipulated to undergo a ‘menses’-like event was collected as described previously (Cousins et al., 2014; Greaves et al., 2014). This tissue was injected into the peritoneal cavity of wild-type (non-luminescent) recipient mice (Greaves et al., 2014) (Fig. 1B). Twenty-one days after intraperitoneal (i.p.) injection of ‘menses’-like endometrial tissue, bioluminescent endometriotic lesions could be detected in the peritoneal cavity of recipient mice following s.c. administration of luciferin (Fig. 1C). When administration routes of luciferin were compared (7 days post injection of tissue), it was noted that i.p. injection in some cases produced a greater bioluminescent signal compared to smaller localized foci following s.c. injection in the same mouse (Fig. 1D). In these animals, unattached material was found in the peritoneal cavity on dissection. This indicates that s.c. administration of luciferin produces bioluminescent signal from attached vascularized implants of endometrial tissue only, and not unestablished ‘floating’ endometrial tissue. Following dissection of lesions at the end of the experiment (Fig. 1E), we validated donor origin by confirming expression of eGFP (Fig. 1F) by endogenous fluorescence imaging and expression of luciferase (Fig. 1G) by immunofluorescence. Lesions exhibited expected histology upon Hematoxylin and Eosin (H&E) staining, comprising of stromal with or without glandular components (Fig. 1H). Thus, we have established bioluminescent non-invasive imaging of lesions in our ‘menses’ mouse model of induced endometriosis that enables clear localization of vascularized lesions. Moreover, the donor origin of lesions can be verified using endogenous fluorescence or immunofluorescence.

### Bioluminescent monitoring in different models reveals variations in lesion luminescence and a gradual resolution of lesions over time

**Fig. 2.**
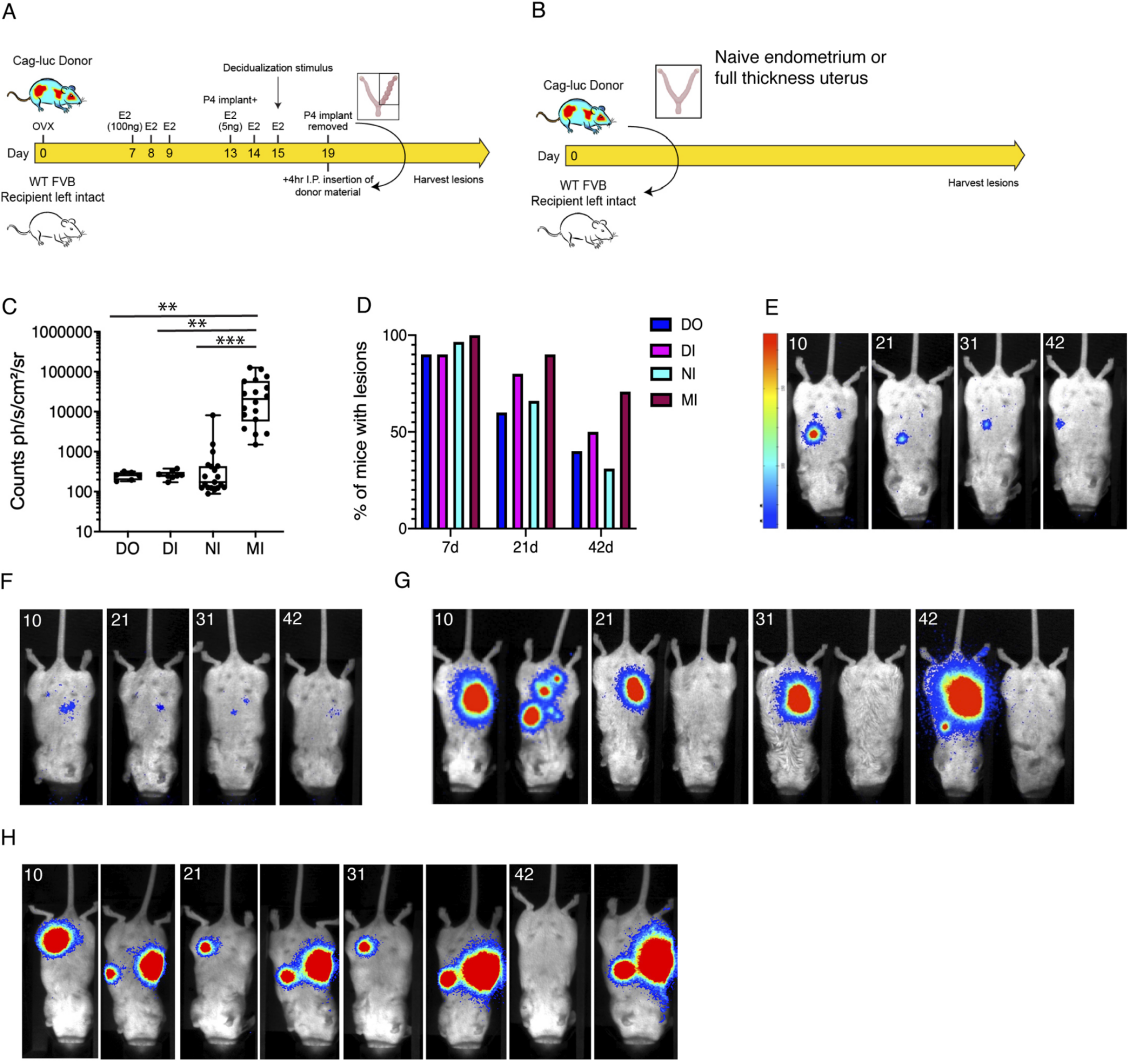
**Comparison of lesion luminescence and longevity across different models of endometriosis.** (A) Schematic representation of the DI mouse model of endometriosis. ‘Menses’-like endometrium from Cag-luc-eGFP was injected i.p. into intact wild-type FVB/N recipient mice. (B) Schematic depicting the minimally invasive mouse models of endometriosis (NI and MI). Naïve endometrium from intact Cag-luc-eGFP was injected i.p. into wild-type FVB/N recipient mice or full thickness uterus (including myometrium) was injected into intact wild-type FVB/N recipient mice. (C) At 21 days post tissue injection the MI model exhibited significantly higher bioluminescent signal compared to all other models (DO, *n*=9; DI, *n*=10; NI, *n*=29; MI, *n*=20). Sample sizes are representative of mice (not lesions) and were achieved by performing experiments 2-4 times. Boxes, interquartile range; whiskers, minimum to maximum. Statistical analysis performed using a one-way ANOVA with Tukey's multiple comparison test (**P<0.01, ***P<0.001). (D) The percentage of mice with focal bioluminescent lesions was quantified at 7, 21 and 42 days post tissue injection. This showed a progressive decline in the number of mice that had detectable lesions in all four models of induced endometriosis. (E-H) Representative longitudinal images of individual mice from day 10-42 post tissue injection. DO model (E); DI model (F); two individual mice from the minimally invasive models (G, NI; H, MI). In G, two mice are presented to illustrate the variation across individual recipients in these two groups. The mouse on the left had a large lesion at day 10 that was maintained at day 42. The mouse on the right had four focal lesions at day 10 that were spontaneously resolved by day 42. In H, two mice are presented. The mouse on the left had one lesion at day 10 that resolved at day 42, whereas the mouse on the right had two lesions on day 10 that progressively increased in size. Bar on left hand side of E (also applies to F-H) is LUT representing photon counts set to 0.0653 and 0.46.

In our established ‘menses’ model of induced endometriosis, recipient mice are ovariectomized and receive oestradiol supplementation (Greaves et al., 2014) [hereafter referred to as ‘DO’ – decidualized (‘menses-like’) endometrium into ovariectomized recipients; Fig. 1B]. The model negates the ability to assess impacts on fertility or therapies that target ovarian signalling, and introduces further complexities in experimental design by requiring the inclusion of surgical sham controls that have also had an ovariectomy. Thus, we chose to adapt the model to use intact recipient mice (referred to as ‘DI’ – decidualized endometrium into intact recipients; Fig. 2A). Additionally, several other variations of endometriosis mouse models exist that vary in the nature of uterine material introduced into the peritoneal cavity. Thus, we compared the two variations of our menses model of endometriosis to a minimally invasive model (Dodds et al., 2017) in which naïve donor endometrium from cycling mice are injected i.p. into intact recipients (hereafter referred to as ‘NI’ – naïve endometrium into intact recipients; Fig. 2B), or full thickness uterine fragments (including myometrium) are injected into recipient mice [‘MI’ model (full thickness including myometrium into intact recipients); Fig. 2B], in our study.

We initially aimed to ascertain any key differences in lesion bioluminescence or longevity originating from the type of donor tissue used to establish endometriotic lesions. At day 21 post-tissue inoculation, the bioluminescent signal intensity was significantly greater in the MI model compared to the NI (*P*<0.001), DI (*P*<0.01) and DO models (*P*<0.05; Fig. 2C). To assess differences in lesion longevity across models, we recorded the number of mice with at least one bioluminescent lesion (when luciferin was administered s.c.) at days 10, 21, 31 and 42 post tissue injection. At day 7 post tissue injection, 90% of mice in the DO and DI group had lesions, 96.6% of mice had lesions in the NI group and 100% had lesions in the MI group. At day 21, 60% of DO mice, 80% of DI mice, 66% of NI mice and 90% of MI mice had bioluminescent lesions (Fig. 2D). We also repeated this analysis at day 42 post tissue injection and identified that 40% of DO mice, 50% of DI mice and only 31% of NI mice still had bioluminescent lesions. In MI mice, 71% still had lesions (Fig. 2D). Longitudinal imaging of mice indicated that in the DO and DI models, lesions exhibited a progressive decline in size, and between 50% and 60% of lesions eventually resolved by 6 weeks post tissue injection (Fig. 2E-H). However, a greater degree of variability in lesion signal intensity was evident in the NI and MI models.

Observation of individual mice over time also revealed that in these alternative models some lesions exhibit spontaneous resolution, whereas others progress in size; we have shown images of two mice from the NI group (Fig. 2G) that clearly illustrate the observed variability within this group – the mouse on the left shows an increase in bioluminescence from day 10 to day 42 (with a newly detectable bioluminescent foci representative of a new lesion at day 42), whereas the mouse on the right has five localized lesions 10 days after tissue injection that resolve by day 21. We observed similar variability in the MI group. In the two representative images presented in Fig. 2H, the lesion detected in the mouse on the left exhibited a gradual decline in size, whereas the two lesions detected in the mouse on the right exhibited an increase in size from day 10 to day 42. In the NI group, we found no difference in the signal intensity of lesions in recipient mice across the oestrus cycle (Fig. S1A), suggesting that one source of variation could be due to the oestrus stage of the donor or the recipient at the time of tissue injection (as previously observed by Dodds et al., 2017). When we separated out the animals based on their oestrus stage, we found that 100% of mice that received oestrus donor endometrium had lesions at day 21 (Fig. S1B). This suggests that lesion establishment/longevity could be greater when oestrus phase donor endometrium is injected; however, the differences are negligible, thus donor endometrial/uterine material from different stages was pooled and divided between recipients going forward in order to reduce any variation. The oestrus stage of the recipient at tissue transfer appeared to have no impact on the number of lesions at day 21 (Fig. S1B).

### Glandular content varies but fibrosis is a consistent feature of lesions collected from different mouse models of endometriosis

**Fig. 3.**
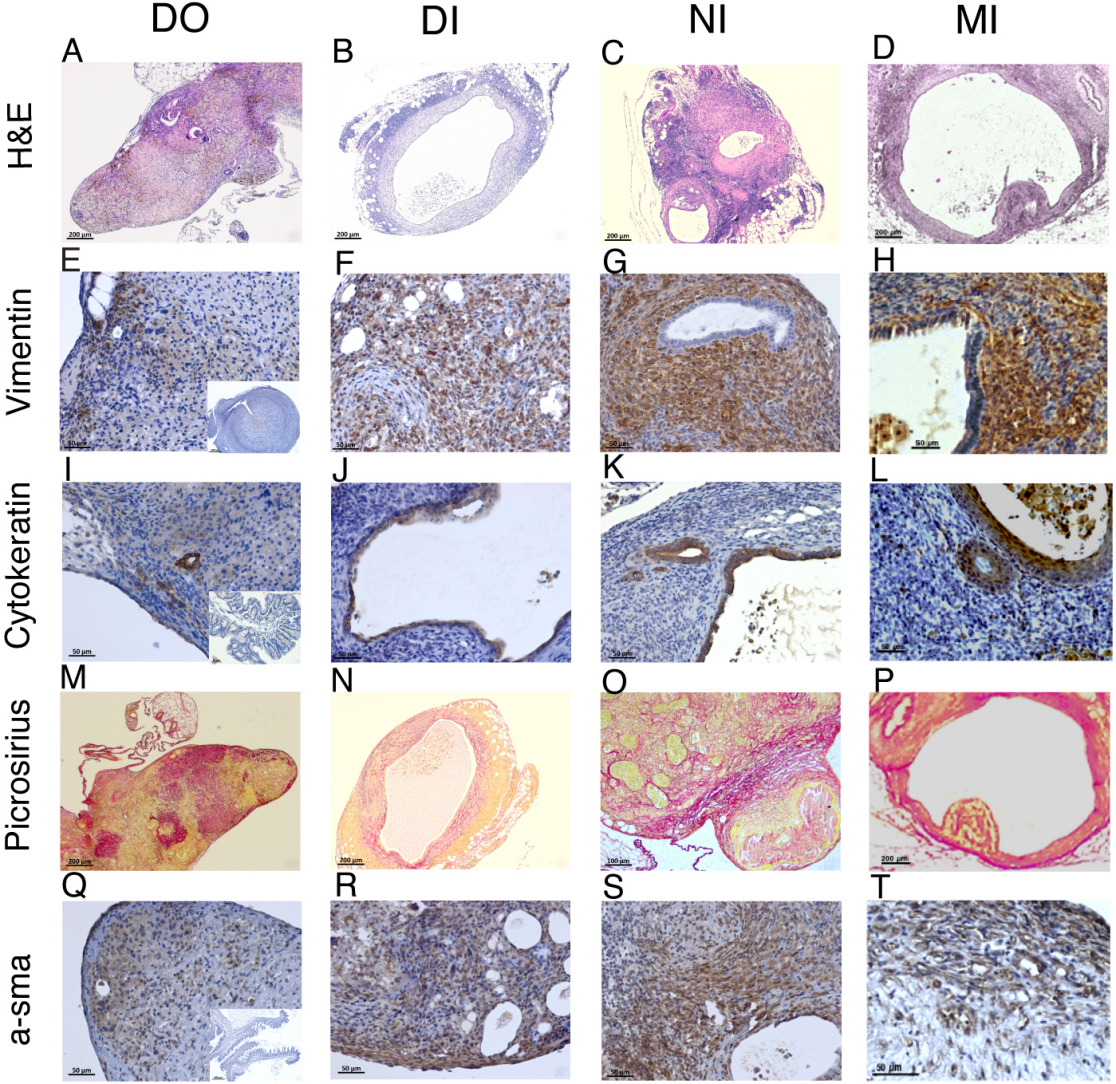
**Identification of endometriosis ‘hallmarks’ in lesions derived from different mouse models of endometriosis.** (A-D) Representative H&E stains from each model. (E-H) Immunodetection of Vimentin to visualize stromal fibroblasts. Inset in E is a negative control section of whole uterus (primary antibody omitted). (I-L) Immunodetection of cytokeratin to visualize epithelial cells. Inset in I is a negative control section of gut (primary antibody omitted). (M-P) PSR stain to identify areas of collagen deposition as a marker of fibrosis. Red stain indicates presence of collagen fibres. (Q-T) Immunodetection of α-smooth muscle actin, a marker of myofibroblasts. Inset in Q is a negative control section of gut (primary antibody omitted). Scale bars: 200 µm (A-D); 50 µm (E-T).

**Fig. 4.**
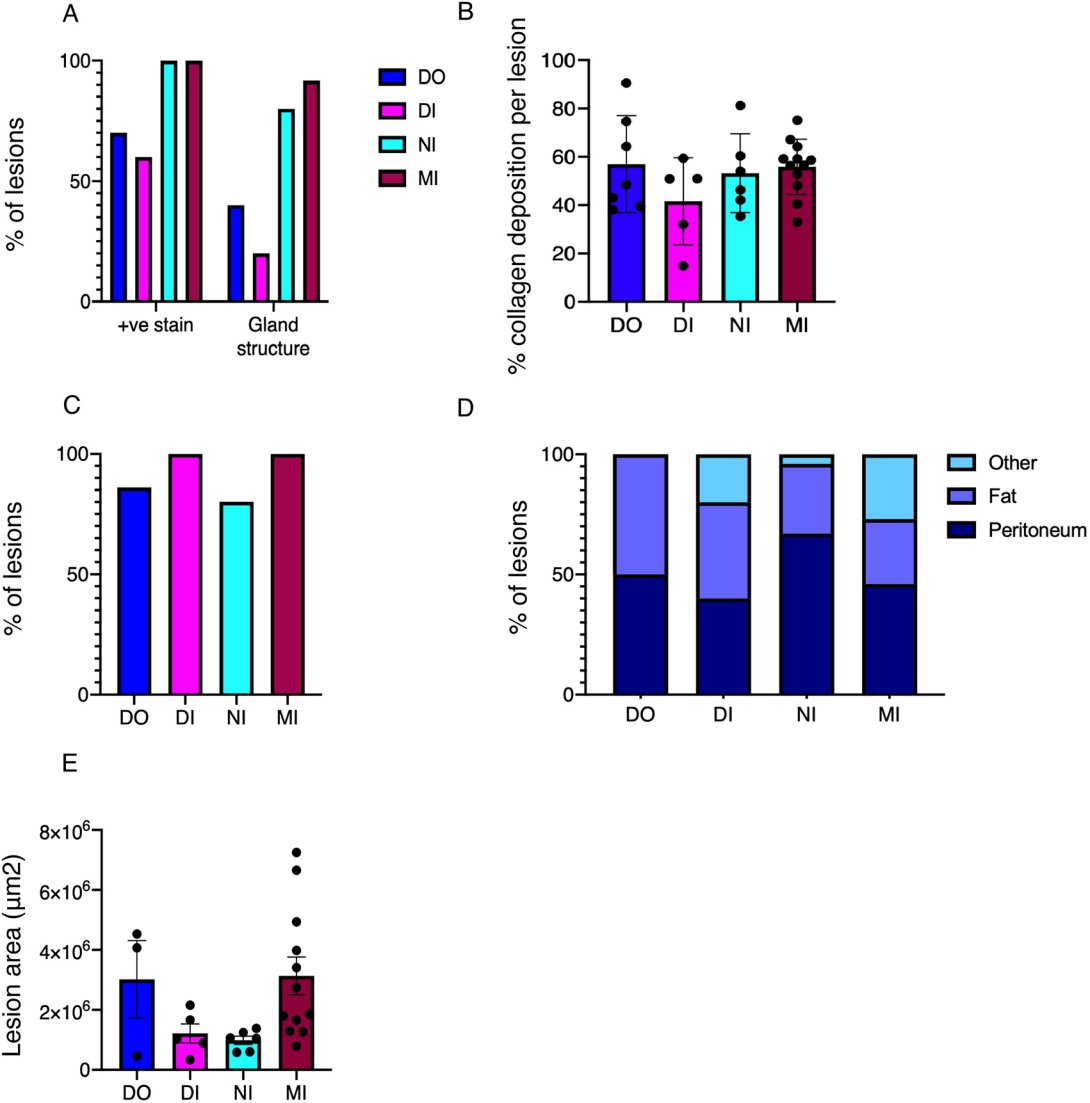
**Quantification of histological structures in lesions recovered at day 42 post tissue injection.** (A) Percentage of lesions that were immunopositive for cytokeratin (irrespective of glandular structure; positive (+ve stain) and the percentage of lesions that exhibited cytokeratin positive glandular structure (DO, *n*=7 lesions; DI, *n*=5 lesions; NI, *n*=5 lesions; MI, *n*=23 lesions). (B) Area of collagen deposition (as a measure of fibrosis) in lesions from the different models. Error bars indicate s.d.; individual data points represent individual lesions. (C) Percentage of lesions that were immunopositive for α-smooth muscle actin (α-sma). (D) Location of lesions recovered from the different models. (E) Area of lesions measured using ImageJ. Error bars indicate s.e.m.; individual data points represent individual lesions.

All biopsies collected from the peritoneal cavity of mice with induced endometriosis (day 42 after tissue injection) were stained with H&E (Fig. 3A-D). At this point, we positively selected samples that exhibited expected lesion architecture (endometrial-like stromal cells with or without epithelial cells). From the DO model, we recovered only three lesions from ten mice (30% in contrast to 40% detectable lesions via imaging). We had four additional lesions from this model and timepoint (from previous experiments) that we could include in the histological analysis in order to increase the sample size to seven lesions. We recovered five lesions from ten mice in the DI model, which was in line with the 50% of mice with detectable lesions via imaging. From the NI model, we recovered only five lesions from 29 mice at day 42 post tissue injection (17.24% in contrast to 31% detectable via imaging). From the MI model, we recovered 23 lesions from 20 mice (71% of mice had detectable lesions at imaging; we recovered lesions from 12 out of 14 mice with positive signal, as well as a further three mice that did not exhibit signal, thus the total recovery was 75%). All positively identified lesions were immunopositive for Vimentin (Fig. 3E-H), a mesenchyme marker, indicating the presence of endometrial-like stromal cells in lesions collected from all four models of endometriosis. We also performed immunodetection for cytokeratin to assess the presence of endometrial-like glandular epithelium (Fig. 3I-L). Initially, we quantified the percentage of lesions that contained cytokeratin-positive glandular structures (see representative images in Fig. 3I-L); in the DO group, 40% of lesions had glands, with 20% in the DI group, 80% in the NI group and 92% in the MI group (Fig. 4A). We also quantified the number of lesions that had cytokeratin-positive cells but without evidence of a glandular lumen. In the DO and DI group, 70% and 60% of lesions, respectively, had cells that were immunopositive for cytokeratin, whereas 100% of lesions from the NI and MI group had cells that were cytokeratin positive (Fig. 4A). To observe the deposition of collagen as a measure of fibrosis in lesions, we performed a Picrosirius (PSR) stain (Fig. 3M-P). All lesions exhibited some areas of dark pink stain (collagen), and when the area of fibrosis in each lesion was quantified, we identified no significant difference across the different models (Fig. 4B). This indicates that fibrosis is a consistent and equal feature in each endometriosis model at the timepoint analyzed. Finally, we performed immunodetection for alpha-smooth muscle actin (α-sma; Fig. 3Q-T), which can be a marker of collagen-producing fibroblasts. In the DO and DI models, 86% and 100% of lesions, respectively, were immunopositive for α-sma. In the NI and MI models, 80% and 100% of lesions were immunopositive for α-sma (Fig. 4C). These results further support the indication that fibrosis is consistent across the models. We also recorded the location from which lesions were recovered (Fig. 4D). In the DO model, lesions were equally distributed between the parietal peritoneal lining and organ-associated fat and mesentery. In the DI model, 20% of lesions were also recovered from other locations, such as on the bladder and outer wall of the uterus. In the NI model, 67% of lesions were recovered from the peritoneal wall, 29% from organ-associated fat and only 4% from other locations. In the MI model, 46% of lesions were recovered from the peritoneum, whereas the remaining lesions were evenly distributed between fat and other locations. There was no significant difference in lesion size across the different models (Fig. 4E).

### Changes in sensory behaviour are evident in different mouse models of endometriosis

**Fig. 5.**
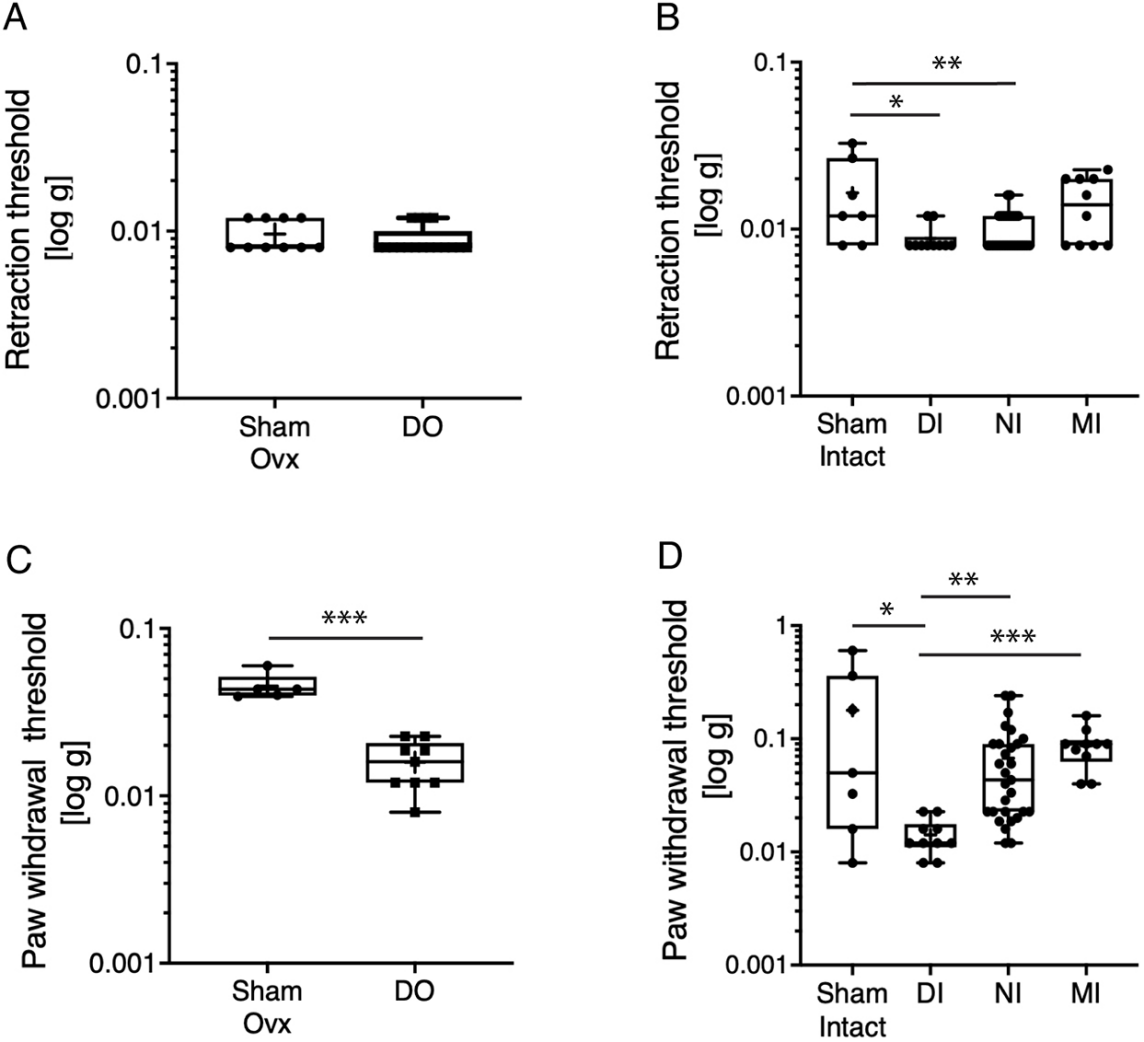
**Changes in sensory behaviour are evident in different mouse models of endometriosis.** (A,B) Mechanical hyperalgesia measured using von Frey filaments applied to the abdomen. Sham-ovx, *n*=10; DO, *n*=17; Sham intact, *n*=7; DI, *n*=10; NI, *n*=29. (C,D) Mechanical hyperalgesia measured using von Frey filaments applied to the hindpaw. Values plotted are an average of measurements taken over 3 days from days 40, 41 and 42. Boxes, interquartile range with mean shown; whiskers, minimum and maximum data points. Statistical significance was determined using Mann–Whitney or Kruskal–Wallis tests (*P<0.05, **P<0.01, ***P<0.001).

We have previously demonstrated that the DO model exhibits mechanical hyperalgesia when von Frey filaments are applied to the abdomen and the hindpaw (Greaves et al., 2017b). In the current study, when von Frey filaments were applied to the abdomen of mice in the DO model, we did not detect a significant difference in abdominal retraction between endometriosis and sham [ovariectomized (ovx) plus oestradiol valerate (EV) plus PBS i.p.] mice (Fig. 5A), contrary to our previous observation. We postulate that this may be due to differences in mouse strain, which in this study was FVB/N but in our previous study was C57Bl/6. In the DI and NI models, mice with endometriosis exhibited significantly lower abdominal retraction thresholds compared to sham animals (*P*<0.05 and *P*<0.01, respectively; Fig. 5B). Abdominal retraction threshold was lower in sham-ovx compared to sham-intact, although this was not statistically significant (Fig. S2A), but along with results shown in Fig. 5A,C, illustrates that sham-ovx mice exhibit changes in sensory behaviour associated with previous surgical procedures, and indicates that intact models are preferable for the assessment of pain-related behaviours. Endometriosis mice in the DO group did exhibit significantly reduced paw withdrawal thresholds compared to sham-ovx (*P*<0.001; Fig. 5C), indicating that referred hyperalgesia consistent with central maladaptation can be detected in this group and is consistent with our previous studies (Greaves et al., 2017b). Paw withdrawal threshold was significantly lower in endometriosis mice from the DI group compared to sham-intact mice (*P*<0.05; Fig. 5D). Mice from the NI and MI groups did not exhibit reduced paw withdrawal thresholds compared to sham-intact mice.

### GnRH antagonism attenuates lesion growth in all intact models but only fully rescues pain response in the NI model

**Fig. 6.**
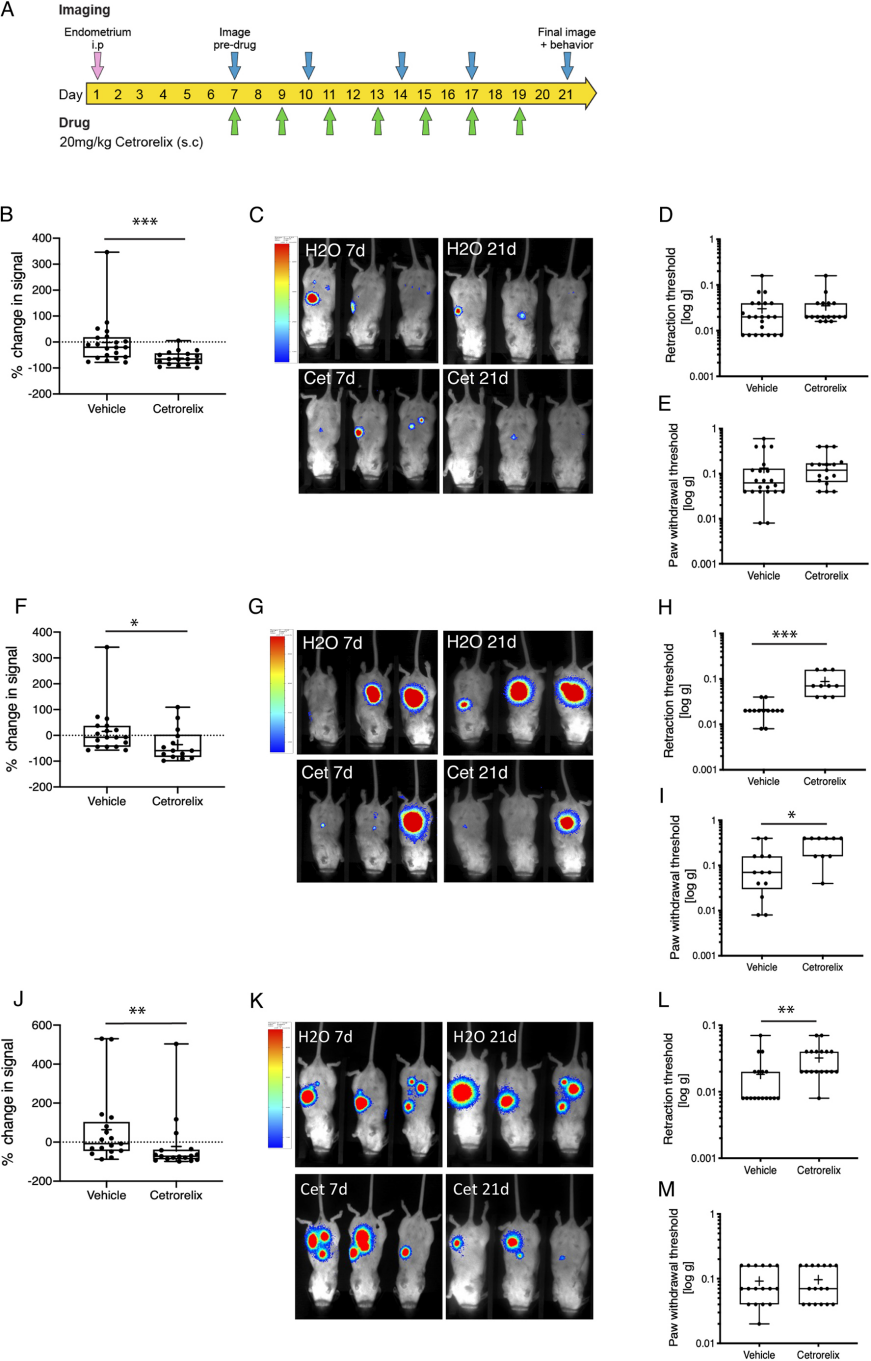
**Response of intact endometriosis models to Cetrorelix.** (A) Schematic showing the treatment and imaging schedule of DI, NI and MI mice. (B) Percentage change in lesion bioluminescent signal calculated from vehicle- (H_2_O) and Cetrorelix (Cet)-treated mice with endometriosis (DI group). Percentage change=(day 21 signal − day 7 signal)/day 7 signal×100. Vehicle, *n*=22; Cetrorelix *n*=17. (C) Representative images from day 7 (pre-drug) and day 21 (end of experiment, DI group). (D) Mechanical hyperalgesia measured using von Frey filaments applied to the abdomen. (E) Mechanical hyperalgesia measured using von Frey filaments applied to the hindpaw. (F) Impact of Cetrorelix treatment on lesion bioluminescent signal intensity (*P*<0.05) in the NI group. Vehicle, *n*=13; Cetrorelix, *n*=10. (G) Representative images from day 7 (pre-drug) and day 21 (end of experiment, NI group). (H,I) Impact of Cetrorelix on mechanical hyperalgesia at the abdomen (H) and hindpaw (I) in the NI model. (J) Impact of Cetrorelix on lesion signal intensity in the MI group. Vehicle, *n*=17; Cetrorelix, *n*=17. (K) Representative images from day 7 and day 21 (MI group). (L,M) Impact of Cetrorelix on mechanical hyperalgesia when von Frey filaments were applied to abdomen (L) and hindpaw (M). Boxes, interquartile range with mean shown; whiskers, minimum and maximum data points. Statistical analysis was performed using a Mann–Whitney test or Kruskal–Wallis test (*P<0.05, **P<0.01, ***P<0.001). Bar on left hand side of C, G and K is LUT representing photon counts set to 0.0653 and 0.46.

Next, we tested the performance of the three intact models in response to a therapy known to attenuate endometriosis symptoms in women. We induced endometriosis in mice, and at day 7 post tissue injection we imaged lesions to obtain a ‘pre-treatment’ baseline. Mice were then randomized into two groups to receive s.c. injection of either vehicle (H_2_O) or GnRH antagonist (Cetrorelix; 20 mg/kg) every 48 h for 2 weeks (Fig. 6A). Administration of Cetrorelix resulted in a significant decrease in ovarian weight (Fig. S2A). Although Cetrorelix reduced lesion signal intensity in the DI model (Fig. 6B,C; *P*<0.001), it did not have a significant impact on pain response (Fig. 6D,E) when von Frey filaments were applied to the abdomen or hindpaw. In the NI model, Cetrorelix attenuated lesion signal intensity (Fig. 6F,G) and pain response when filaments were applied to both the abdomen (Fig. 6H; *P*<0.001) and hindpaw (Fig. 6I; *P*<0.05). Cetrorelix also reduced lesion signal intensity in the MI model (Fig. 6J,K; *P*<0.01). Local pain response was attenuated when von Frey filaments were applied to the abdomen (Fig. 6L; *P*<0.01), but there was no difference in referred pain hyperalgesia when von Frey filaments were applied to the hindpaw (Fig. 6M).

## DISCUSSION

In our current study, we have assessed the utility of bioluminescent imaging in our existing mouse model to allow us to make repeated measurements of lesion number and longitudinal size. We then compared our existing model to three different variations of mouse models of induced endometriosis in order to compare differences in bioluminescent signal, lesion histology, pain-related behaviour and response to a cogent therapy: Cetrorelix (GnRH antagonist). Our main findings were fivefold: (1) lesion signal intensity was significantly greater in the MI model (full thickness uterine fragments in intact mice); this also translated to a greater number of lesions per mouse. (2) A gradual resolution of lesions was evident in all models; however, in the NI and MI models, some lesions exhibited evidence of progression in size and occasional development of additional bioluminescent foci, suggesting the formation of new lesions. (3) The presence of glandular epithelia in lesions varied across models, with more detected in the NI and MI models, whereas the presence of fibrosis was consistent. (4) The degree of changes in sensory behaviour varied across models; the most significant changes were evident in the DI model, in which robust sensitization was observed at the abdomen and hindpaw. (5) Administration of Cetrorelix decreased lesion bioluminescent signal intensity in all models, but its ability to reduce changes in sensory behaviour varied between models. Taken together, these data demonstrate evident differences between mouse models that need to be carefully considered during the design of *in vivo* experiments.

The finding that many lesions gradually decrease in size over time and ∼50% spontaneously resolve by 6 weeks, whereas a small proportion of mice in the NI and MI models exhibit progression of bioluminescent foci, was intriguing as this is also known to occur in non-human primates and women. Resolution was evident when recipients were ovariectomized and supplemented with oestradiol or left with their ovaries intact, suggesting this phenomenon occurs regardless of the hormonal status of mice. Endometriotic lesions are known to evolve during active disease, as evidenced by the changing colour of lesions over time. Baboons with experimentally induced endometriosis that were subject to multiple laparoscopies demonstrated a large proportion of red active lesions shortly after disease induction, which changed colour to blue, chocolate, white and mixed pigmentation as the disease progressed (Harirchian et al., 2012; Hastings and Fazleabas, 2006). Spontaneous resolution of lesions was also evident in the baboon model, with only 41% of the initial lesions identifiable at the end of the study (15 months post inoculation). However, at 12 months after tissue inoculation, 51% of lesions present were newly detected lesions, indicating that in baboons, endometriosis can be progressive (Harirchian et al., 2012). Observational studies in women also suggest that lesions are dynamic. Second-look laparoscopy studies reported by Sutton et al. (1997) indicated that of the patient population studied, 29% showed some regression of disease, whereas the remaining majority progressed or remained stable.

Thus, our modelling experiments in mice do mirror the fluctuating nature of ectopic tissue to a certain extent, although increased levels of resolution and lower levels of progression are evident in mice with induced endometriosis. This finding can be extrapolated to a new concept: that instead of modelling ‘endometriosis’, in a large proportion of mice we are in fact modelling a ‘healthy’ response to refluxed endometrial tissue. This can be exploited as it allows rationale to be developed on processes that might be defective in women with endometriosis. It remains unknown what is different about the lesions that progress or are maintained long-term, and future work should draw comparisons between resolving versus non-resolving mice. Recently, we identified a ‘protective’ population of monocyte-derived large peritoneal macrophages (LpM) in our menses mouse model of endometriosis that limit the development of lesions (Hogg et al., 2021). Using gain- and loss-of-function experiments, we demonstrated that depletion of this population increases development of lesions, whereas reprogramming the peritoneal niche such that embryo-derived LpM are ablated and the niche is repopulated afresh with monocyte-derived LpM, leads to decreased lesion development. These data support the idea of immune dysfunction in women with endometriosis (Ahn et al., 2015), which requires further exploration. The resolving versus non-resolving platform can also be exploited to further define this peritoneal immune environment in response to continued, progressive or resolved endometriosis.

In the NI and MI mouse models we occasionally visualized progressive lesions (either new foci or growth of lesions). This suggests that the ‘menses-like’ material used to inoculate recipient mice in the DO and DI models may not contain the cell types required to establish chronic or progressive endometriosis. The cellular origins of endometriosis have yet to be fully elucidated (Filby et al., 2020). However, it has been postulated that endometrial stem/progenitor cells are the cells of origin of endometriosis lesions (Gargett, 2007; Cousins et al., 2018). Previous studies in a mouse model of endometrial breakdown and repair have identified that only 0.14% of decidual cells are label-retaining cells prior to breakdown (Kaitu'u-Lino et al., 2012), suggesting that very few putative stromal stem-like cells are transferred into recipient mice in the DO and DI models. Evaluation of menstrual blood collected from women with and without endometriosis suggests that mesenchymal stem-like cells and epithelial progenitors are present in menstrual effluent, with a trend towards increased progenitors in women with endometriosis (Masuda et al., 2021). This highlights that there are disparities in progenitor populations shed in women versus mice induced to menstruate. A two-stem/progenitor cell hypothesis has also been proposed whereby both stromal and epithelial progenitor cells are required to form typical endometriosis lesions with both stromal and epithelial compartments (Wang et al., 2020). Based on the results presented in the current study, we postulate that a higher proportion of both stromal and epithelial progenitors are present in the donor material in the NI and MI models, as lesions recovered from these models exhibit higher levels of histologically confirmed glandular epithelia, and these models show some progression, whereas the other models do not. Interestingly, we found that a greater number of mice had lesions at day 21 when oestrus stage endometrium was introduced into the peritoneal cavity. It has recently been shown that the number of stem-like cells fluctuates across the oestrus cycle in mice and that an elevated number can be detected at the oestrus stage (Singh and Bhartiya, 2021), thus adding further evidence for a role for stem cells. However, despite pooling tissue from different oestrus stages to reduce variability, we still only observed progression in a few mice. Thus, at present, we are unable to conclude exactly what determines chronic lesions or those prone to progression or resolution. In the models that use donor ‘menses’ endometrium (DO and DI), decidualization is artificially induced (Greaves et al., 2014). However, women with endometriosis reportedly exhibit impaired decidualization, with stromal fibroblasts present in menstrual effluent also demonstrating an impaired ability to decidualize when cultured *in vitro* (Warren et al., 2018). One possible explanation for increased resolution in the DO and DI models is that decidualized stromal fibroblasts may exhibit a lower propensity to form chronic lesions, although this requires further investigation.

It is also important to highlight that the variability of lesions containing glandular structures is consistent with clinical findings that not all lesions possess evident epithelia. These lesions are often diagnosed as ‘stromal endometriosis’ (Clement, 2007). The variability in the occurrence of fundamental lesion components across the models reflects the heterogeneity of lesions found in women and may have key implications for response to therapy. For example, ‘stromal endometriosis’ may respond differently to ‘traditional’ lesions that possess epithelia. To this end, it has been proposed that treatment resistance in endometriosis could be associated with the evident fibrosis present within lesions, and this component (Groothuis and Guo, 2018) may require alternative treatment, possibly in combination with hormone therapy.

It is striking that despite controlling for the amount of tissue transferred per animal, the establishment, longevity and signal intensity of lesions in the MI model far outperform the other models. However, despite this model exhibiting traditional lesion histology (robust evidence of glandular structures in all lesions), the impact on pain response is minimal compared to the DI model. This has led us to speculate that perhaps the different models could represent different subtypes of lesions. The DO and DI models that use ‘menses-like’ endometrium to initiate lesions may represent an inflammatory subtype, such as red active lesions. The finding that sensory behaviour modifications were more pronounced in the DI model, and that Cetrorelix was not able to rescue these changes, further substantiates this idea and that this model could be compared to endometriosis that is refractory to hormonal treatment in women. However, it has been reported that red peritoneal lesions contain proliferative glandular epithelia (Nisolle and Donnez, 1997), whereas a limited number of lesions from the DO and DI models contain cytokeratin-positive gland structures. One limitation of the current study is that we only used a measure of evoked mechanical hyperalgesia to assess sensory behaviour, and should we have included further assessments, the MI model may have exhibited a more severe sensory phenotype. To this end, the non-surgical model published by Fattori et al. (2020) (using full thickness uterus as donor material) exhibited abdominal mechanical pain, as well as spontaneous pain-related behaviour. The mice did not exhibit thermal hyperalgesia or any differences in dynamic weight bearing (Fattori et al., 2020). Recently, a mouse model of deep infiltrating endometriosis has been established, in which mice with induced endometriosis were infused with the neurotransmitter substance P and calcitonin gene-related peptide via osmotic pumps. The lesions recovered from mice exhibited hallmarks of deep infiltrating lesions, including the presence of endometrial epithelial and stromal cells, an abundance of fibromuscular content, encapsulation in surrounding tissues or organs and extensive fibrosis. The mice also exhibited thermal hyperalgesia in response to the hotplate test (Yan et al., 2019) but spontaneous behaviour was not recorded. Future studies should aim to incorporate a wide range of behavioural tests such that parallels between studies can be drawn more easily.

Cetrorelix is a preferable hormone therapy for endometriosis compared to previously used GnRH agonists because fewer side effects occur and no oestradiol add-back is required (Finas et al., 2006). A study including sequential laparoscopic and clinical evaluation before and after Cetrorelix therapy in women with endometriosis revealed a reduction in disease stage for women with minor endometriosis (Küpker et al., 2002). Regression occurred in 60% of cases; however, in women with severe [ASRM (American Society for Reproductive Medicine) stage IV] endometriosis, Cetrorelix had no impact (Küpker et al., 2002). In the present study, all three intact mouse models exhibited a reduction in lesion bioluminescent signal intensity and a visible reduction in bioluminescent foci following treatment with Cetrorelix. Despite this desirable effect on lesions, attenuation of mechanical hyperalgesia was only observed in the NI and DI models, highlighting disparities in response to this established therapy for the treatment of endometriosis-associated pain.

**Fig. 7.**
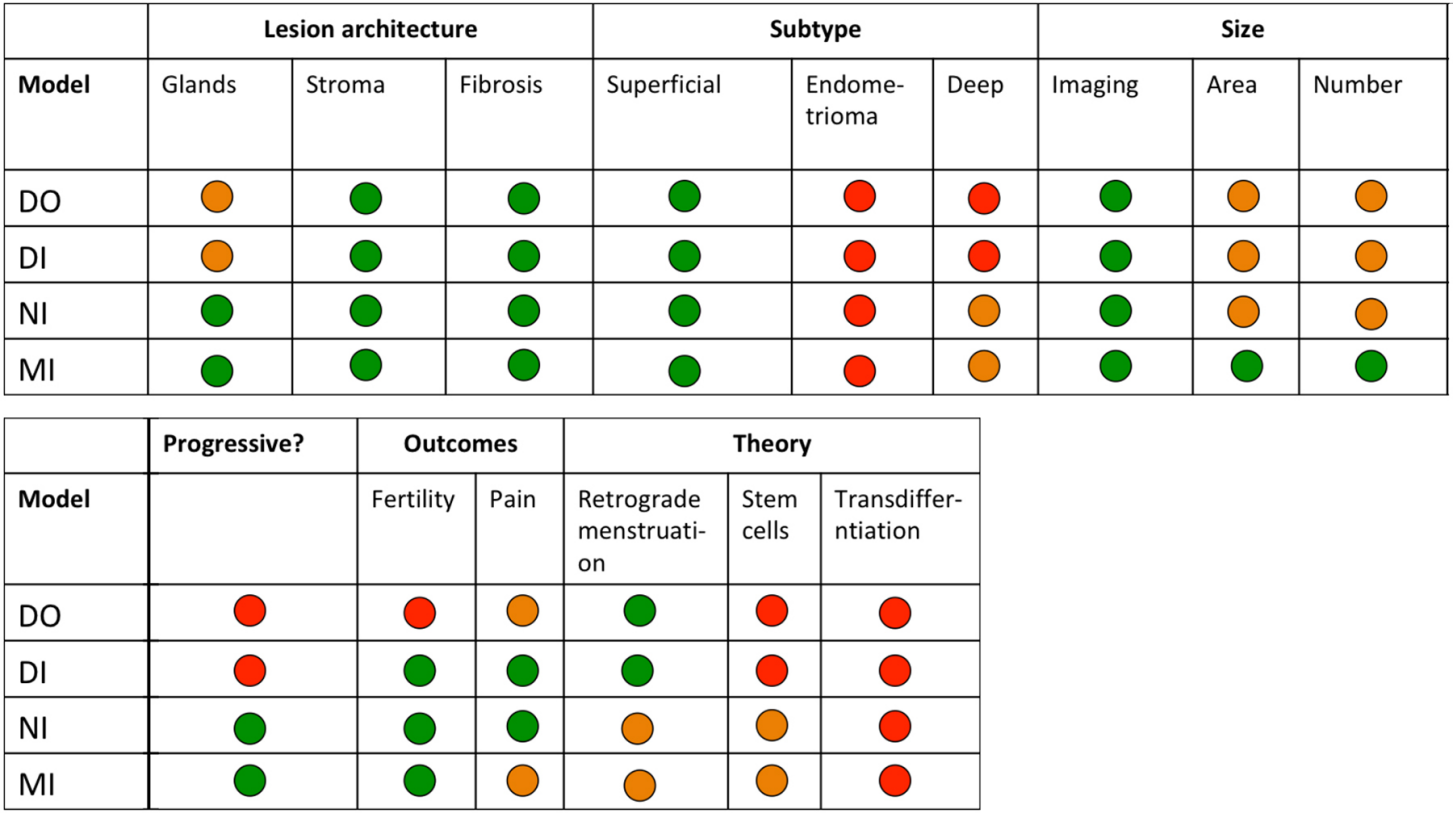
**Blueprint of models compared in the study.** We have used a traffic light scoring system to denote whether a model performs well (green), partially (orange) or not at all (red) in modelling a particular feature or outcome. We have scored the intact models as green for measuring fertility outcomes because they have the ability to be used in this context; however, we have not yet demonstrated any differences in pregnancy outcome in the models.

We have proposed a blueprint that summarizes the features and utilities of the different mouse models analyzed in this study (Fig. 7). The DO and DI models that use ‘menses-like’ endometrium for the formation of lesions aim to recapitulate the physiological process of retrograde menstruation; however, they perform less well in displaying the traditional lesion architecture of well-developed glandular structures. The DI and NI demonstrate the most significant changes in sensory behaviour, whereas only the NI and MI exhibit a progressive phenotype in some mice. We have not observed ovarian or deep infiltrating lesions in any of the models. However, a genetically engineered model that mimics the natural spread of invasive endometrium, including the formation of lesions on the ovary, has been published recently (Wilson et al., 2020). Taken with our findings that include a predicted inflammatory phenotype (the ‘menses’ model) that might be refractory to hormonal suppression and the above-mentioned model of deep infiltrating endometriosis (Yan et al., 2019), the field now has a toolbox of models that may be compared to different subtypes of endometriosis and could be used in combination to test promising innovative treatments for endometriosis.

Our studies highlight variability in the performance, and possibly the underlying ‘disease’ mechanisms, of different mouse models of induced endometriosis. Currently, we do not know enough about the pathophysiology and ontogeny of endometriosis to be able to suggest a standardized model. Rather, the evident variability in models might also reflect different subtypes and heterogeneity of response in women. Thus, we suggest that there is a requirement for preclinical testing in multiple ‘models’ to ascertain how robust a potential treatment might be in targeting endometriosis, and we call for international collaboration in the testing of potential therapies across institutes and models in order to generate robust preclinical data that may have greater success in clinical studies.

In summary, we have presented data indicating that not all mouse models of induced endometriosis are equal and that the nature of the ‘donor’ uterine material is an important indicator for the presence of epithelial glandular structures and long-term maintenance/progression of induced lesions. Importantly, we have demonstrated that using ‘menses-like’ endometrium produces transient lesions that exhibit a high degree of resolution by 6 weeks, although the hyperalgesia that develops in this group is more severe compared to the other models and is also resistant to GnRH treatment (although lesion bioluminescence is reduced). We propose that the differences evident in each of the models reflect the heterogeneity/subgroups observed in women with endometriosis and may reflect discrete differences in disease mechanisms and aetiology. These findings are vital for the field of discovery science and preclinical testing of potential therapeutics in mouse models of induced endometriosis, and support the concept of testing therapies in multiple models and may help in identifying subgroups that respond/do not respond to particular therapies.

## MATERIALS AND METHODS

### Animals and reagents

FVB-Tg(CAG-luc,-GFP)L2G85Chco/J (stock number 008450|L2G85) were purchased from The Jackson Laboratory (Bar Harbor, ME, USA), and were bred and maintained in specific pathogen-free facilities at the University of Edinburgh and the University of Warwick. A breeding stock of wild-type FVB mice was maintained to produce experimental cohorts of recipient mice, or bought from Charles River (UK). All experiments were permitted under licence by the UK Home Office and were approved by the University of Edinburgh and University of Warwick Animal Welfare and Ethical Review Bodies. Mice had access to food and water *ad libitum*. Ambient temperature and humidity were 21°C and 50%, respectively. To visualize bioluminescent endometriosis lesions, the substrate D-luciferin (1.5 mg/100 μl in PBS; Sigma-Aldrich, Dorset, UK) was injected s.c. prior to imaging. Female mice between the age of 8 and 12 weeks were used for the experiments. Mice with induced endometriosis were administered 20 mg/kg Cetrorelix acetate in dH_2_O s.c. (Sigma-Aldrich) every 48 h. The dose was selected based on previous *in vivo* studies (Danforth et al., 2005; Otto et al., 2012). For the Cetrorelix experiments, mice were randomly allocated to either vehicle or drug group, and the investigator performing von Frey testing was blinded to the experimental group.

### Endometriosis modelling in mice

#### ‘Menses’ mouse model of endometriosis

Endometriosis was induced in mice as described previously (Greaves et al., 2014). Briefly, donor CAG-luc-GFP mice were ovariectomized and exposed to a hormone schedule of oestradiol followed by oestradiol plus progesterone and decidualization stimulus. Progesterone withdrawal was then initiated to induce endometrial breakdown and shedding akin to menstruation (Cousins et al., 2016). The decidualized endometrial mass was scraped away from the underlying myometrium, resuspended in saline, passed through an 18-gauge needle once and injected into the peritoneal cavity of wild-type FVB/N mice (bred in-house or bought commercially). Recipient mice were subject to ovariectomy (ovx) and oestradiol supplementation (500 ng oestradiol valerate twice weekly). We refer to these mice as ‘DO’ (decidualized endometrium into ovx recipients; *n*=10). A group of sham animals were included for behavioural assessment (*n*=5). These animals had undergone ovx and received the same oestradiol supplementation as endometriosis mice. Shams were subjected to an i.p. injection of saline instead of endometrial tissue. We also used ‘menses’ endometrium to induce endometriosis in intact mice (‘DI’, *n*=10). Intact shams for this group were injected with saline instead of tissue (*n*=7). The amount of endometrial tissue used to inoculate the peritoneal cavity in each model was standardized to ∼40 mg.

#### Minimally invasive mouse models of endometriosis

We also used two variations of the minimally invasive mouse model of endometriosis (Dodds et al., 2017). In the first version, endometrium was dissected away from the myometrium using sharp dissection and fragments (∼40 mg tissue) were resuspended in saline, passed through an 18 g needle once and then injected into intact recipients (*n*=29; ‘NI’). Oestrus stage was determined in donors and recipients by vaginal smear and retrospective cytological analysis (McLean et al., 2012). In the second version, whole uterus was sliced into small fragments and resuspended in saline (∼40 mg), and passed through an 18 g needle prior to i.p. injection into intact recipients (‘MI’; *n*=20). Sham animals (*n*=7) were included for behavioural assessments. These animals received i.p. injection of saline instead of endometrial tissue.

#### *In vivo* optical imaging of luciferase activity and bioluminescence quantification

Following anaesthesia with isofluorane, mice were injected s.c. with 1.5 mg luciferin potassium salt. Following a 5 min incubation to allow the luciferin to be circulated and a plateau of bioluminescent activity to be reached, mice were imaged using a PhotonIMAGER (Biospace Lab, Paris, France). Luminescence level was measured in regions of interest (ROIs) corresponding to the pelvis, abdomen and torso combined. We used the same ROI across experiments and captured bioluminescent activity on the front and then the back of each mouse for 7 min. A reading of photons/s/cm^2^/sr was calculated using M3 Vision software (Biospace Lab), background reading was subtracted and then the reading from the front and the back of the mouse averaged.

### Behavioural assessment

Mechanical hyperalgesia in mice with induced endometriosis was measured using calibrated Semmes–Weinstein von Frey filaments (Stoelting, Wood Vale, IL, USA), according to the manufacturer's instructions and as described previously (Greaves et al., 2017b). Briefly, mice were allowed to acclimatize to the apparatus and then filaments were applied perpendicular to the abdomen or plantar surface of the hindpaw in ascending order. The weight of the filament that caused a withdrawal reflex in 50% of applications was recorded. The investigator performing the measurements was blinded to the experimental group. Behaviour assessment was performed on days 40, 41 and 42 post tissue injection, and the mean reading across the 3 days was plotted for each mouse. For the Cetrorelix studies, readings were taken on days 19, 20 and 21, and the mean recording was plotted for each mouse.

### Histology and immunodetection

Following fixation, mouse lesions were processed into 5-μm sections on slides and stained using H&E. Only lesions containing identifiable stroma with or without glandular epithelium were used for further analysis. For analysis of collagen deposition by Picro Sirius Red (PSR) stain, sections were de-waxed in xylene and rehydrated. Sections were incubated with PSR dye for 2 h and then washed, dehydrated and cleared in xylene prior to mounting. Single colour immunohistochemical analysis was performed according to standard protocols and as described previously (Greaves et al., 2014). In brief, citrate antigen retrieval and blocking of endogenous peroxidase activity in 3% H_2_O_2_ was performed. Non-specific epitopes were blocked using a species-specific serum blocking solution (1:4 serum in Tris-buffered saline plus 5% bovine serum albumin). Sections were incubated with appropriate primary antibody (see Table 1; Chung et al., 2019; Yuri et al., 2015; Martinez et al., 2019a) overnight at 4°C in a humidified chamber. Sections were then incubated with species-specific horseradish peroxidase (HRP)-conjugated secondary antibody (ImmPRESS, Vector Labs) for 30 min at room temperature. Antibody staining was visualized by incubating sections with 3,3-diaminobenzidine for 10 min, followed by dehydration, clearing in xylene and mounting. Imaging was performed using a Zeiss Z1 Imager microscope or EVOS cell imaging system (Thermo Fisher Scientific). All antibodies were previously validated and the specificity of the secondary antibody was confirmed in our experiments using negative controls (omission of primary antibody).

**Table 1.**
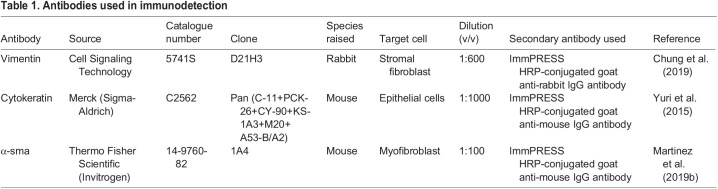
Antibodies used in immunodetection

### Quantification of Picro Sirius Red stain

Analysis of collagen deposition was performed using ImageJ. The image scale was set and the image cropped and the background removed. The ‘select’ tool was used to draw around the lesion outline and measure the area of the lesion. The image was then converted to an ‘RGB’ channel stack. The green channel was used to set the threshold so that only the area stained red with PSR was quantified. The area of collagen deposition (red stain) was then calculated as a percentage of the total lesion area.

### Statistical analysis

Mechanical hyperalgesia measurements generate the most variation, thus sample sizes were determined in our previous experiments that detected a statistically significant difference in withdrawal threshold between sham and endometriosis mice (Greaves et al., 2017b). Normality of data was assessed using Shapiro–Wilk and Kolmogorov–Smirnov tests. The data did not pass normality tests and so non-parametric analyses were performed. Statistical analysis was performed using a Kruskal–Wallis with Dunn's multiple comparison test when comparing more than two groups, and a Mann–Whitney test was used for comparing two groups only.

## Supplementary Material

Supplementary information
