# Supplementary material for: Bioluminescent imaging in induced mouse models of endometriosis reveals differences in four model variations
Source: Dis Model Mech. 2021 Aug 31;14(8):dmm049070. doi: 10.1242/dmm.049070 (PMC8419713; doi:10.1242/dmm.049070)
Supplement: Supplementary information [file dmm-14-049070-s1.pdf]

A

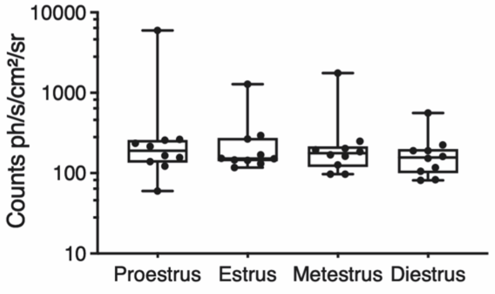

B

Mice with lesions @ d21 – stage of the estrus cycle (donor)			
Proestrus	Estrus	Metestrus	Diestrus
5/9	6/6	3/6	5/8
Mice with lesions @ d21 – stage of the estrus cycle (recipient)			
Proestrus	Estrus	Metestrus	Diestrus
5/9	2/4	5/6	7/10

Fig.S1. Impact of estrus stage on lesion signal intensity and longevity.

A. Lesion signal intensity remains comparable across the estrus cycle in the NI model. Vaginal cytology was performed on the day of imaging in order to determine if estrus stage has any impact on lesion growth / bioluminescent signal intensity.

B. Impact on estrus stage of donor and recipient mice on lesion longevity. Vaginal cytology was performed on donor mice the day uterine material was collected for transfer to recipient mice. Vaginal cytology was also performed on recipient mice on the day of endometrial tissue receipt. 100% of mice that received estrus stage endometrium still had lesions on d21 post tissue injection.

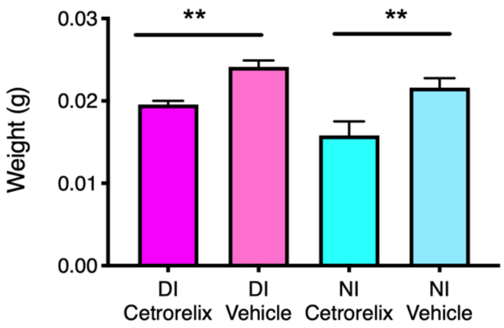

Fig.S2. Ovarian weight is significantly decreased in mice treated with Cetrorelix.

A. Ovary weights are significantly decreased in Cetrorelix treated mice with induced endometriosis (vehicle n=10, Cetrorelix n=100. Statistical testing was performed using a one-way ANOVA and Tukey’s multiple comparison test. **:p<0.01.
